# Oxidative Stress: A Major Player in Cerebrovascular Alterations Associated to Neurodegenerative Events

**DOI:** 10.3389/fphys.2018.00806

**Published:** 2018-07-03

**Authors:** Cristina Carvalho, Paula I. Moreira

**Affiliations:** ^1^CNC - Center for Neuroscience and Cell Biology, University of Coimbra, Coimbra, Portugal; ^2^Institute for Interdisciplinary Research, University of Coimbra, Coimbra, Portugal; ^3^Laboratory of Physiology, Faculty of Medicine, University of Coimbra, Coimbra, Portugal

**Keywords:** neurovascular unit, endothelial cells, oxidative stress, mitochondria, NADPH oxidases, Alzheimer’s disease

## Abstract

The brain is one of the most exquisite organs in the body with high metabolic demands, and requires a tight regulation of the surrounding environment. This tight control is exerted by the neurovascular unit (NVU) comprising different cell types, where endothelial cells play the commander-in-chief role. Thus, it is assumable that even slight perturbations in NVU might affect, in some cases irreversibly, brain homeostasis and health. In this line, recent findings support the two-hit vascular hypothesis for neurodegenerative conditions, where vascular dysfunction underlies the development of neurodegenerative diseases, such as Alzheimer’s disease (AD). Knowing that endothelial cells are rich in mitochondria and nicotinamide adenine dinucleotide phosphate (NADPH) oxidases, two major reactive oxygen species (ROS) sources, this review aims to gather information on how oxidative stress is in the front line of vascular alterations observed in brain aging and neurodegenerative conditions, particularly AD. Also, a brief discussion about the therapeutic strategies aimed to protect against cerebrovascular diseases is included.

## Introduction

The brain integrates and regulates several central and peripheral signals to maintain body homeostasis (Ronnett et al., 2009). So, it is not surprising that the brain is an organ with a high energy demand, although it represents only 2% of the body weight (Mergenthaler et al., 2013). In fact, it is widely known that proper neuronal activity entails high amounts of energy. However, the capability of the brain to store energy is very reduced, requiring a constant supply of energy substrates, namely glucose, through blood flow to fulfill its energy needs (Ohta et al., 1992). For that reason the brain receives about 15% of cardiac output and accounts for 20% of total body oxygen consumption (Moreira et al., 2009; Nunomura et al., 2009; Ronnett et al., 2009). At this point, it is worth mentioning the neurovascular coupling, where neurons, glial cells and blood vessels communicate to each other to regulate cerebral blood flow (CBF) and vessels permeability depending on location and neuronal activity in order to efficiently maintain energy substrates supply to satisfy the metabolic needs (Gordon et al., 2007). Cerebral blood vessels comprise unique properties forming the blood–brain barrier (BBB), a physical barrier that permits the passage of water, some gases and lipophilic molecules by passive diffusion and the selective transport of certain molecules (e.g., glucose) and protects against external toxins and pathogens. Thus, even slight alterations in BBB properties can be responsible for the onset/progression of neurological diseases. From all the BBB constituents (endothelial, mural and glia cells, astrocytes and macrophages), endothelial cells that form blood vessels, play a major role in BBB proper functioning (Daneman and Prat, 2015). Blood vessels control the influx and efflux transport allowing, for example, the entry of glucose and amino acids from the blood into the central nervous system (CNS) and the removal of specific waste products from the CNS into the blood (Daneman and Prat, 2015). More recently, Kubíková et al. (2017), performed a mapping of brain microvessels obtained from two healthy human adult brain samples and found that in certain brain areas microvessels density is higher than others, which can reflect the different susceptibilities to vascular damage. Even slight alterations in brain vasculature can underlie different neurodegenerative events. Indeed, it is widely described that conditions interfering with brain vasculature, such as stiffening of cerebral arteries or increased vessel tortuosity, caused by diabetes and hypertension among other conditions, can induce BBB breakdown underlying the development of neurodegenerative conditions such as Alzheimer’s disease (AD) (Carvalho et al., 2010, 2013, 2014; Steinman et al., 2017). It is also known that in many cases BBB integrity is deeply affected by oxidative stress. In fact, increased reactive oxygen species (ROS) production contribute to endothelium dysfunction and increased permeability of BBB (Enciu et al., 2013). These alterations are mainly attributed to the redistribution and/or altered expression of critical tight junction proteins such as claudin-5 and occludin (Schreibelt et al., 2007; Lochhead et al., 2010).

Although it is widely accepted that vascular changes play a crucial role in neurodegenerative diseases, none of the available therapies are effective when translated to clinical trials revealing some gaps in the mechanisms behind the vascular and tissue brain changes under pathological conditions. Thus, studies aimed to develop new non-invasive techniques to better understand why and when changes occur bring a new hope for the treatment of conditions characterized by cerebrovascular alterations (Vaas et al., 2017). In the next section we will discuss the role of oxidative stress in brain vascular alterations putting the focus on mitochondria and nicotinamide adenine dinucleotide phosphate (NADPH) oxidase (Nox).

## Major Players in Vascular Oxidative Stress

It is widely known that cells homeostasis depends on the regulated levels of ROS. Low/moderate levels of ROS can act as signaling molecules, which are crucial to maintain normal cells function, while uncontrolled generation of ROS causes oxidative damage contributing to cells dysfunction and damage (Chen et al., 2017).

**FIGURE 1:**
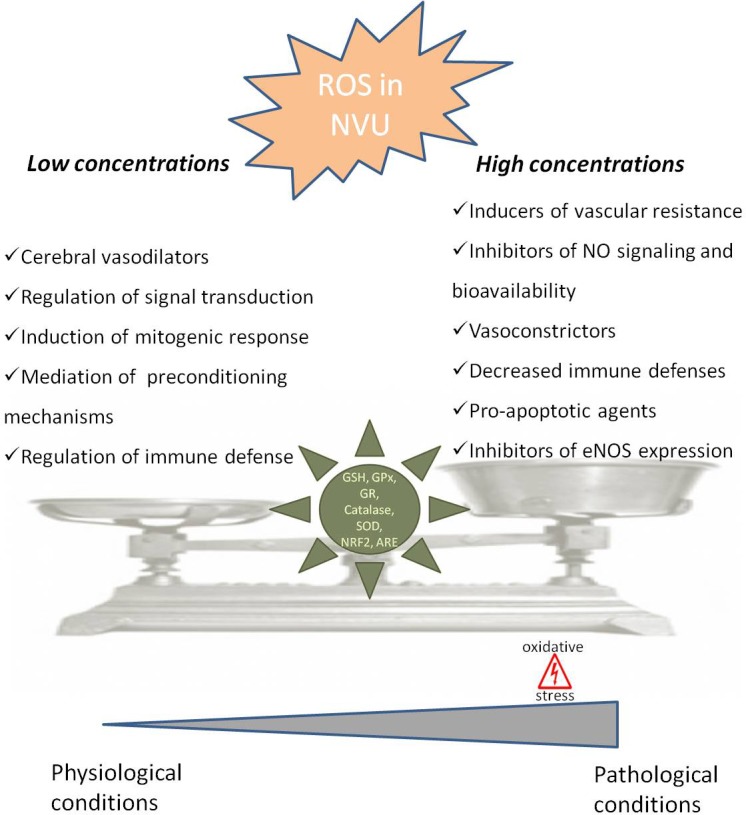
Dual role of reactive oxygen species in neurovascular unit. In neurovascular unit (NVU) reactive oxygen species (ROS) play a controversial role with low/moderate levels of ROS acting as signaling molecules, which are crucial to maintain normal cells function, while uncontrolled generation of ROS causes oxidative damage contributing to cellular dysfunction and cells damage. In physiological situations ROS can act as cerebral vasodilators, regulators of signal transduction, inducers of mitogenic response and immune defense mechanisms, regulators of blood flow and effectors of preconditioning mechanisms. However, when ROS concentrations pass a certain threshold, they lead to an increase in blood flow resistance, decreased nitric oxide (^•^NO) bioavailability, decreased vasodilatation and immune response, increased apoptosis and decreased endothelial nitric oxide synthase (eNOS) expression, leading to pathological conditions. The delicate balance of ROS levels is regulated by cellular antioxidant systems such as glutathione (GSH), glutathione peroxidase (GPx), glutathione reductase (GR), superoxide dismutase (SOD), catalase, nuclear factor erythroid 2-related factor 2 (NRF2) and antioxidant response element (ARE).

Physiological ROS levels can play important roles in cerebral vasculature (De Silva and Miller, 2016). Studies performed in animal models show that ROS can contribute to the regulation of brain perfusion through their action in vascular tone control (Miller et al., 2009; Grochowski et al., 2018). Indeed, physiological levels of ROS play a major role as cerebral vasodilators (**Figure 1**). For example, it has been shown that the addition of NADPH, the substrate for Nox, in cerebral vessels *in vitro* and *in vivo* cause hydrogen peroxide (H_2_O_2_)-dependent vasodilatation (Didion and Faraci, 2002; Park et al., 2004; Miller et al., 2005; De Silva and Miller, 2016).

The neurovascular unit (NVU), containing neurons, astrocytes, pericytes, microglia and endothelial cells, is equipped with a powerful antioxidant defense systems that include glutathione (GSH), glutathione peroxidase, glutathione reductase, superoxide dismutase and catalase (Tayarani et al., 1987; Halliwell, 2001). GSH in particular has been shown to play an important role in the maintenance of BBB integrity (**Figure 1**) (Agarwal and Shukla, 1999). Also, the nuclear factor erythroid 2-related factor 2 (NRF2) seems to play a major defense role by modulating microglial dynamics (Rojo et al., 2010), by protecting astrocytes and neurons from toxic insults (Lee et al., 2003; Vargas and Johnson, 2009) and by regulating the expression of antioxidant enzymes (Shah et al., 2007; Yan et al., 2008). Additionally, NRF2 induces secondary defense proteins via interaction with the antioxidant response element (ARE) in the promoter region of target genes (**Figure 1**). Interestingly, astrocytes have a higher capability of efficiently increase GSH and ARE-linked gene expression, which allows astrocytes to be more protected than neurons against moderate levels of oxidative stress (Vargas and Johnson, 2009).

A failure in the ability of NVU cells to maintain the proper balance between ROS production and their neutralization causes the disruption of NUV and brain homeostasis predisposing to neurodegenerative conditions (Carvalho et al., 2013; Wevers and de Vries, 2016). It has been shown that following an injury microglia and astrocytes produce high levels of ROS via Nox, which seem to have harmful effects in the expression of important molecules involved in BBB integrity (e.g., ZO-1, claudin-5 and occluding). Pericytes, which are in close proximity to endothelial cells, play a vital role in the integrity of BBB (Armulik et al., 2010). Under pathological conditions, pericytes are highly susceptible to oxidative stress (Shah et al., 2013) resulting from overproduction of mitochondrial ROS (Shah et al., 2013). Moreover, it has been demonstrated that ROS production by activated microglia causes pericytes apoptosis (Ding et al., 2017). However, there is a lack of information regarding the role of ROS in pericytes under physiological conditions. Endothelial cells signaling seems to be crucial in the regulation of NVU proper functioning with increased ROS formation playing a major role in the alteration of NVU function and coupling (Girouard and Iadecola, 2006; Faraco et al., 2016).

It was also shown that ROS-induced changes in microcirculation can have profound implications in brain vascular pathophysiology due to alterations in blood flow resistance and, consequently, in regulation of blood pressure (**Figure 1**) (Staiculescu et al., 2014). Both superoxide (O_2_^•-^) and H_2_O_2_ are able to cause both relaxation as well as contraction of cerebral blood vessels depending on the concentration and presence of other species (Faraci and Sobey, 1998; Allen and Bayraktutan, 2009; Freeman and Keller, 2012). In fact, oxidative stress can impair cerebral vascular function via the disruption of endothelium-dependent nitric oxide (^•^NO) signaling (**Figure 1**) (Girouard et al., 2007; Mayhan et al., 2008; Miller et al., 2010b). It is widely described that the reaction of O_2_^•-^ with ^•^NO leads to a decrease in ^•^NO bioavailability and, consequently, a decrease in its vasodilator, anti-proliferative, and anti-inflammatory properties (De Silva and Miller, 2016). Furthermore, increased levels of H_2_O_2_, a common vasodilator in cerebral circulation, can act as a pro-apoptotic agent in cerebral vascular cells (**Figure 1**) (Li et al., 2003). Increased levels of ROS can also lead to an increase in Rho kinase signaling (Aghajanian et al., 2009) interfering with endothelial nitric oxide synthase (eNOS) expression and activity, affecting ^•^NO production (**Figure 1**) (Faraco et al., 2013).

Considering vascular cells, there are several ROS sources such as mitochondrial electron-transport chain, cyclooxygenases (COXs), lipoxygenases, cytochrome P450 reductases, xanthine oxidase, nitric oxide synthase (NOS) and Nox (Miller et al., 2010a). However, in this review, we only discuss mitochondria and Nox, both of them widely described as main producers of ROS either in physiological and pathological conditions in endothelial cells.

### Mitochondria: More Than an Energy Producer

Reporting to the history of biology evolution, mitochondria are described as organelles derived from aerobic bacteria that in ancient times invaded proto-eukaryotic cells as parasites (Dromparis and Michelakis, 2012). From there, a symbiotic relationship evolved with mutual benefits and mitochondria became intracellular organelles (Dromparis and Michelakis, 2012). Although mitochondria are widely described as energy producers through the highly conserved oxidative phosphorylation (OXPHOS) process (Cadenas and Davies, 2000; Chan, 2006), nowadays the role of mitochondria reached substantially higher importance in cell homeostasis due to their involvement in several vital processes such as cell growth and differentiation, cell cycle control and death (Osellame et al., 2012; Carvalho et al., 2015), intermediary metabolism, calcium (Ca^2+^) homeostasis and signaling, and apoptosis (Chan, 2006).

However, mitochondrial energy production can be a double-edge sword. Indeed, OXPHOS is not 100% efficient and during this process an electron leak occurs between mitochondrial complexes leading to the production of ROS such as O_2_^•-^, ^•^NO, hydroxyl radical (HO^•^), peroxynitrite (ONOO^-^), and H_2_O_2_ (Richter and Kass, 1991; Ricquier and Bouillaud, 2000; Goetz and Luch, 2008). As previously mentioned, low/moderate ROS levels exert a beneficial role by activating protective mechanisms (Valko et al., 2007; Correia et al., 2010; Alfadda and Sallam, 2012). However, when ROS levels reach critical values they lead to oxidative stress and activate anomalous signaling mechanisms that can lead to cells degeneration and death (Brown and Borutaite, 2001; Sheu et al., 2006).

Brain endothelial cells seem to possess a number of mitochondria higher than that observed in peripheral endothelial cells (Oldendorf et al., 1976; Alyautdin et al., 2014). However, in comparison with other cell types with higher energy requirements, mitochondria content in endothelial cells is modest. In rodent models, mitochondria compose 2–6% of the cell volume as opposed to 28% in hepatocytes and 32% in cardiac myocytes (Dromparis and Michelakis, 2012; Kluge et al., 2013). These observations support the idea that mitochondria is not a major source of energy in brain endothelial cells. In fact, several studies support the idea that brain endothelial cells obtain a large proportion of their energy from anaerobic glycolytic metabolism of glucose (Spahr et al., 1989; Mertens et al., 1990; Culic et al., 1997). Actually, mitochondria are more likely to serve primarily as essential signaling organelles in the vascular endothelium (Quintero et al., 2006; Tang et al., 2014; Busija et al., 2016).

The activation of mitochondria by physiological stimuli or pharmacological agents leads to the liberation of vasoactive factors by the endothelium, which exert a major role in the modulation and maintenance of BBB integrity and brain homeostasis (Busija and Katakam, 2014). Moreover, it is far known that aging leads to a decrease in mitochondria number in cerebral endothelial cells associated with the loss of BBB integrity (Mooradian, 1988). The BBB high demand of mitochondrial activation comes with a price, the prospect for an increased ROS production that in physiological conditions is regulated by antioxidant enzymes such as glutathione reductase, manganese superoxide dismutase, catalase, among others (Freeman and Keller, 2012). Additionally, it has been demonstrated that vascular endothelial growth factor (VEGF) exerts its functions in endothelial cells migration through mitochondrial ROS (Wang et al., 2011), a process involved in several physiological processes such as wound healing and vascular repair (Freeman and Keller, 2012). Moreover, studies in human coronary resistance arteries showed that mitochondrial ROS are involved in endothelium regulation of vascular homeostasis (Liu et al., 2003). In the same study, the authors reported that mitochondrial ROS have an influence in vascular regulation and health. Indeed, the authors tested mitochondrial complexes I and III and Nox inhibitors and observed that only the mitochondrial inhibitors were able to exert effects in the regulation of flow-induced dilation (Liu et al., 2003). Besides the above evidence more studies are needed to explore the role of mitochondrial ROS in cerebral vasculature during disease progression, in contrast to the extensive literature concerning systemic vessels.

### NADPH Oxidases

The Nox family is also broadly studied due to its main catalytic function of ROS production by transference of an electron to molecular oxygen (Takac et al., 2012). The Nox family is composed by different isoforms: Nox1 to 5, Duox1 and Duox2 with a broad expression in different organs and tissues and with different cellular locations at vascular walls (de Almeida et al., 2017). Except for Nox-5 and DUOX-1 and -2, Nox are phagocytic oxidases, whose main task is to generate ROS to kill foreign pathogens at homeostasis. Of note, the major source of endothelial cells ROS comes from NOX-1, -2, -4, and -5 (Pendyala et al., 2009; Drummond and Sobey, 2014). Different Nox isoforms seem to produce different forms of ROS; Nox1 and 2 mainly produce O_2_^•-^; Nox4 mainly produces H_2_O_2_ and Nox5 seems to produce both O_2_^•-^ and H_2_O_2_ (Dikalov et al., 2008; Helmcke et al., 2009). Under physiological conditions, Nox-derived ROS seem to exert an important role in the regulation of vasodilatation (Togliatto et al., 2017). Although it is considered that under physiological conditions Nox activity is constitutively low, when its function increases with the consequent increase in ROS production, it can trigger ROS production by other sources (Landmesser et al., 2003). Indeed, it has been described that Nox-derived ROS play a major role in coordinating some physiological processes such as innate immunity, modulation of redox-dependent signaling cascades, and can act as cofactors in the production of hormones (Drummond et al., 2011). Furthermore, it is described that H_2_O_2_ produced by Nox family can exert an endothelium-derived hyperpolarizing role causing vasodilatation and reducing blood pressure in mice (Ray et al., 2011). The Nox activity depends on the stimuli, such as cytokines (De Keulenaer et al., 1998), growth factors (Brandes et al., 2001), hyperlipidemia, and high glucose (Jansen et al., 2013). Additionally, Nox family is known for its role as oxygen sensors, modulating the different responses to hypoxia through hypoxia-inducible factor 1 alpha (HIF-1α) mRNA induction and HIF-1α stabilization (Gorlach et al., 2001), a process that is responsible for alterations in gene expression due to the oxidation of target proteins.

However, the overproduction of ROS by Nox family plays a major role in disruption of vascular homeostasis, which can underlie the development of neurodegenerative diseases. Of notice, so far only Nox2 and Nox4 were associated with endothelium dysfunction probably due to their major role as vascular ROS producers, in comparison to the other Nox isoforms (Matsuno et al., 2005; Takac et al., 2012).

Compelling evidence shows that Nox-derived oxidative stress causes many of the deleterious effects of angiotensin II on the cerebral vasculature. It was observed that angiotensin II acutely and chronically, increases O_2_^•-^ production by Nox in rodent cerebral vessels (Girouard et al., 2006, 2007; De Silva and Faraci, 2016). Also, functional alterations on rodents’ cerebral arterioles following angiotensin II treatment are prevented by co-treatment with the ROS scavenger MnTBAP and the Nox2 peptide inhibitor gp91ds-tat (Girouard et al., 2006). It was also reported that angiotensin II can also activate and increase O_2_^•-^ production in cerebral vessels by activating Nox1 (Jackman et al., 2009).

Interestingly, although not extensively studied, the Nox family activation and expression seems to be seasonal, a fact that could be correlated with seasonal alterations in oxidative stress and endothelium dysfunction observed in humans and rats unveiling a new strategic pathway to future studies of clinical relevance (Hopkins et al., 2011; Konior et al., 2014).

## Aging and Neurodegenerative Disorders: When Things Start to Fail

The aging process causes several alterations in brain blood vessels such as decreased elasticity, increased cerebrovascular remodeling and calcification, gradual cerebrovascular wall stiffness, low-grade and widespread inflammation and oxidative stress (Camici et al., 2015), factors that increase the risk for cerebrovascular diseases. So far, there is no agreement of how aging process occurs although several theories can be found in the literature. One of the most accepted theory for aging was first proposed by Harman (1956) and is known as “free radical theory of aging,” which postulates that ROS levels increase with age, being responsible for deoxyribonucleic acid (DNA), proteins and lipids oxidative damage (Harman, 1956).

**FIGURE 2:**
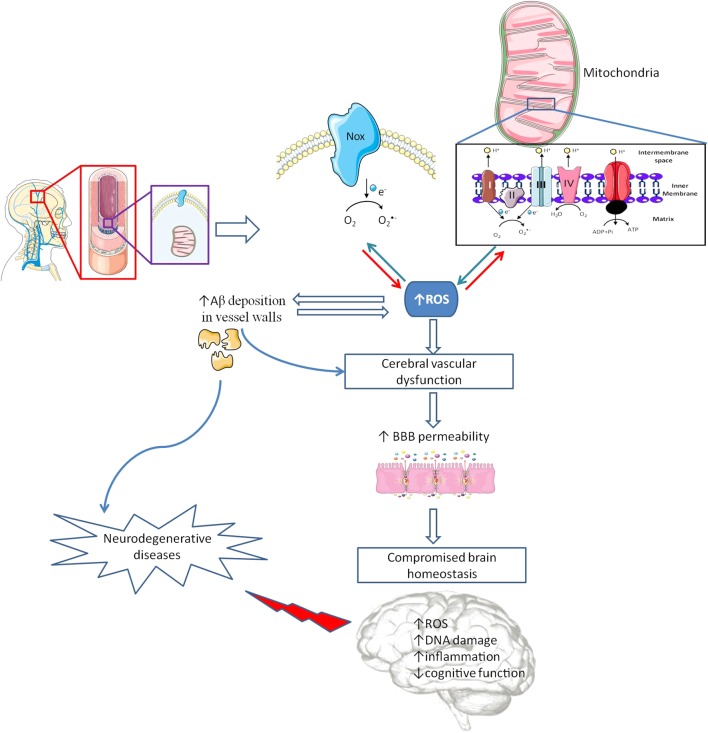
Cerebrovascular alterations and brain damage. The brain is a very delicate organ that relies on an extremely controlled environment and on a constant energy supply due to its high metabolic demand, which depend on blood–brain barrier (BBB) proper functioning. Besides other cell types (astrocytes, pericytes, microglia and a basement membrane made from structural proteins) endothelial cells (EC) assume the major role in controlling BBB integrity. Inside EC, mitochondria and nicotinamide adenine dinucleotide phosphate (NADPH) oxidases (Nox) act as main sources of reactive oxygen species (ROS). In pathological conditions, an increased ROS levels cause cerebrovascular dysfunction, which promotes an increase in BBB leakage interfering with brain energy supply and homeostasis, and increasing amyloid β (Aβ) peptide deposition in vascular walls. Alterations in BBB features are associated with oxidative stress and damage and inflammatory processes, among other deleterious alterations, contributing to cognitive defects and, eventually, the development of neurodegenerative diseases.

Recent findings demonstrated the existence of BBB permeability alterations during normal aging in human hippocampus using advanced dynamic contrast-enhanced magnetic resonance imaging (DCE-MRI) (Montagne et al., 2015). It is also known that aging increases BBB susceptibility to different challenges and its permeability is less controlled allowing the entrance of neurotoxic substances, which affect brain homeostasis (Zlokovic, 2011; Caplan et al., 2017). In fact, BBB dysfunction is more pronounced under pathological conditions, including in mild cognitive impairment (MCI) individuals, supporting the idea that vascular alterations are early events in cognitive deficits (**Figure 2**) (Montagne et al., 2015). Using a multiphoton microscope, Han et al. (2015) observed increased levels of O_2_^•-^ in the cerebral vessels of aged PS1/APP transgenic mice, a model of AD. Also, electron and confocal microscopy studies revealed that in F344 rats aortas, the expression of the mitochondrial biogenesis factors, such as mitochondrial transcription factor A and peroxisome proliferator-activated receptor-γ coactivator-1-alpha (PGC-1α), decreases with aging (Ungvari et al., 2008). Moreover, an increase in mitochondrial H_2_O_2_ production was observed in aged arterial rat vessels leading to an activation of nuclear factor-κB (NF-κB) and induction of inflammatory phenotypic changes in aged vasculature (Ungvari et al., 2007). Furthermore, an age-dependent decline in mitochondrial complexes I, III, and IV subunits expression was observed in rat aortas, although the authors did not specify if all the subunits of the three complexes were evaluated or not (Ungvari et al., 2008). Also, Wenzel et al. (2008) were able to show an age-dependent increase in mitochondrial ROS production and mitochondrial DNA lesions causing aorta vascular dysfunction in two different mice models, the ALDH-2–/– and MnSOD–/– models, which are deficient in aldehyde dehydrogenase-2 (ALDH-2) and manganese superoxide dismutase (MnSOD), respectively. Furthermore, the *in vitro* use of mitochondrial complex I inhibitor rotenone, after angiotensin II-induced O_2_^•-^ production, showed significant improvements in endothelial dysfunction and tolerance in human aortic endothelial cells (Dikalov et al., 2014; Mikhed et al., 2015). Furthermore, the use of preconditioning strategies as a therapeutic intervention is emerging and under extensive scrutiny. Indeed, studies from our laboratory were able to show that treating rat brain endothelial cells with non-lethal cyanide concentrations, which lead to increased mitochondrial ROS production, was able to confer protection against *a posterior* high glucose-mediated damage, preventing apoptotic cell death (Correia et al., 2012). Importantly, Correia et al. (2012) were able to prove that in the absence of mitochondrial DNA, using mitochondrial-depleted DNA human teratocarcinoma NT2 cells, this effect was abrogated, emphasizing mitochondrial role in endothelium protection by preconditioning (Correia et al., 2012). Moreover, the use of mitochondrial-targeted antioxidants such as MitoTempo, showed promising results in counteracting age-related and microgravity-induced and hyperglycemia-induced endothelial dysfunction (Carvalho et al., 2014; Zhang et al., 2014). Indeed, Mitotempo was able to protect endothelial primary cultures of diabetic mouse model (db/db) and rat cerebral arteries against amyloid β (Aβ) toxicity (Carvalho et al., 2014) and lead to improvements in spatial working memory and motor skill learning in young 5xFAD mouse models of AD (Lu et al., 2015). Moreover, it was also able to recover mitochondrial dysfunction observed in rat cerebral arteries of animals exposed to microgravity conditions through reduction of mitochondrial ROS levels, increased mitochondrial potential and improvement in mitochondrial respiratory chain function (Zhang et al., 2014).

Mitochondrial-derived oxidative stress can also activate vascular inflammation in aged carotid arteries and vessels (Csiszar et al., 2007; Ungvari et al., 2007) leading to atherosclerosis through activation of endothelial NF-κB, which is responsible for the upregulation of adhesion molecules and increased monocyte adhesiveness in aged aortic arteries (Ungvari et al., 2007). Moreover, changes in Nox activity and/or expression is widely described in aged brain vessels (Park et al., 2007; Mayhan et al., 2008). Nevertheless, the mechanistic role of Nox in the aging process remains obscure (Sahoo et al., 2016).

Data from the literature also show that in cultured aged cerebromicrovascular endothelial cells (CMVECs) the mRNA expression levels of Nox2 and 4 subunits were upregulated compared with young CMVECs, a phenomenon similar to that observed in the myocardium, leading to increased levels of oxidative stress (Toth et al., 2014). Although further studies must be performed in order to corroborate this hypothesis, it seems that, similarly to the myocardium, in CMVECs the increased expression of Nox 2 and 4 causes oxidative stress, which activates the renin–angiotensin–aldosterone system (RAAS) (Wang et al., 2010). This activation can occur through a ROS-induced increase in angiotensinogen (AGT), a 60-kDa α2-globulin glycoprotein that constitutes the precursor of RAAS; stimulation of renin, the enzyme responsible for the initiation of the RAAS pathway; and release or regulation of angiotensin converting enzyme (ACE) activity, crucial for the formation of angiotensin II, the major effector of RAAS (Morato et al., 2017). Furthermore, since Nox 4 is usually localized in mitochondria, its increased expression can also contribute to increased levels of mitochondrial ROS potentiating the aging process as well as age-related diseases (Sahoo et al., 2016). The use of Nox inhibitors showed promising results in reducing oxidative stress levels and in improving endothelial function in several disease models (Girouard et al., 2006; Kahles et al., 2007; Matsumoto et al., 2007; Miller et al., 2010a). Also the use of resveratrol seems to be effective in restoring cerebromicrovascular endothelial function through a downregulation of Nox-derived ROS production in aged mice (Toth et al., 2014).

Despite the extensive literature stating that age-associated oxidative stress can lead to cerebrovascular dysfunction and cognitive decline, there is a significant gap in our knowledge regarding the exact mechanisms underlying these defects (Tarantini et al., 2017a).

## Cerebrovascular Oxidative Stress and Neurodegenerative Events: the Case of Alzheimer’s Disease

Although the previous alterations seem to be common features in physiological aging of vessels, somewhere over the way those defects can become more pronounced triggering neurodegenerative events (Kling et al., 2013). Indeed, recent reports suggest a major role for the NVU in the initiation of neurodegeneration (Nelson et al., 2016). In fact, NVU dysfunction leads to an increased BBB permeability with the subsequent entry of neurotoxic molecules into the brain disturbing its homeostasis. A decrease in the removal of neurotoxic substances from the brain, and a deficient nutrient delivery system eventually culminate in neuronal loss and synaptic dysfunction (Zlokovic, 2008). Thus, a decline in cerebrovascular function in age-associated diseases, such as AD, is easily understandable (Girouard and Iadecola, 2006). Indeed, it is estimated that about 60–90% of AD patients also present cerebrovascular alterations including cerebral amyloid angiopathy (CAA), microinfarcts and ischemic lesions and microvascular degeneration (Jellinger and Mitter-Ferstl, 2003; Bell and Zlokovic, 2009).

It has been recently reported the existence of BBB and vascular alterations in several diseases such as Parkinson’s disease (PD), Huntington’s disease and cerebral small vessel disease (Lin et al., 2013, 2015; Lee and Pienaar, 2014; St-Amour et al., 2015; Al-Bachari et al., 2017) although there is a gap concerning the mechanisms underlying those alterations and the role of vascular oxidative stress in these pathologies.

Concerning AD, the most common form of dementia in the elderly, more information exists about the vascular alterations that occur in this disease (Gallart-Palau et al., 2016). Several clinical and basic evidence points to the existence of a major contribution of both large artery and small vessel disease in the pathogenesis of AD (Iadecola, 2013; De Strooper and Karran, 2016; Faraci, 2017). Having this into account, the two-hit vascular hypothesis of AD emerged, postulating that cerebrovascular damage is an initial insult that is self-sufficient to initiate neuronal injury and neurodegeneration, but can also promote accumulation of Aβ peptide, a neurotoxic peptide, in the brain (Nelson et al., 2016). Thus, events that compromise vascular health can initiate a cascade of deleterious events that culminate in AD development. In fact, previous studies from our laboratory demonstrated that some risk factors for AD, such as hyperglycemia, increase the susceptibility of brain endothelial cells to Aβ peptide, a phenomenon related to ROS overproduction (Carvalho et al., 2014). Moreover, several genetic [apolipoprotein E4 (APOE4), phosphatidylinositol-binding clathrin assembly protein (PICALM), clusterin, presenilin 1, amyloid precursor protein (APP), mesenchyme homeobox gene 2 (MEOX2)] (Nelson et al., 2016) and non-genetic (hypertension, metabolic syndrome, hypercholesterolemia, atherosclerosis, alcohol and substance abuse, among others) (Kivipelto et al., 2002; Skoog and Gustafson, 2006; Nelson et al., 2016; Campos-Pena et al., 2017) risk factors for AD seem to be linked with alterations in vascular function supporting the idea that vascular alterations play a major role in AD pathology.

Recent studies in wild type mice showed that, even under normal conditions, there is a different regional susceptibility of the cerebrovasculature to oxidative stress (Austin et al., 2015). Vessels from cortex and hippocampus, the two main areas compromised in AD, contain significantly higher levels of intracellular O_2_^•-^ and increased protein levels of both Nox 2 and 4 (Austin et al., 2015). Other studies revealed significant changes in blood vessels morphology, decreased vascular density and increased vessels tortuosity in AD brains (Hunter et al., 2012). Moreover, the use of recent technologies such as arterial spin labeling magnetic resonance imaging (MRI), functional blood-oxygen-level-dependent (BOLD)-MRI, fluorodeoxyglucose-positron emission tomography (FDG-PET), and single-photon emission computerized tomography (SPECT), showed decreased levels of CBF in early phases of human AD progression (Daneman and Prat, 2015). Recently, Lourenço et al. (2017) showed that reduced CBF changes found in triple transgenic for AD (3xTg-AD) mice preceded memory dysfunction thus suggesting that cerebrovascular dysfunction could be the primary cause of neurovascular uncoupling in AD. Those observations are supported by MRI, transcranial doppler and SPECT studies revealing different patterns of decreased CBF coincident with the brain regions where neuropathological alterations are more relevant in AD (Hu et al., 2010; Pimentel-Coelho and Rivest, 2012). Those alterations seem to be closely related with the levels of oxidative stress in the cerebrovasculature. Studies from our laboratory showed that 3xTg-AD mice present an increased BBB permeability, in cortex and hippocampus (Carvalho et al., 2013), these alterations being correlated with decreased aconitase activity, an enzyme whose activity is inhibited by O_2_^•-^ (Carvalho et al., 2013). The increased levels of ROS appeared to be the result of impaired activity of mitochondrial enzymatic complexes I–III (**Figure 2**). Moreover, and similarly to our observations in mitochondria isolated from whole brain of 3xTg-AD mice (Carvalho et al., 2012), Golgi silver impregnation studies using multiple samples from the hippocampus and cortices of AD patients showed that brain endothelial cells present enlarged mitochondria, disruption of the mitochondrial cristae and reduced abundance (Baloyannis and Baloyannis, 2012).

An interesting fact is that even though mitochondrial morphology is very similar in male and female rodent cerebral arteries, major differences can be observed concerning mitochondrial protein mass, respiration, and function (Rutkai et al., 2015), which is in accordance with sex/gender differences observed in several brain-related studies (Giordano et al., 2013; Christov-Moore et al., 2014; Ingalhalikar et al., 2014; Sun et al., 2015) including the susceptibility to develop AD (Candeias et al., 2017).

Mitochondrial ROS overproduction also increases Aβ deposition in vessels walls, which is a common feature in many cases of AD (**Figure 2**). Indeed, 34–35% of human AD cases present CAA, predominantly in the occipital lobe (Love et al., 2014). Aβ deposition in vessels walls seems to initiate a vicious cycle, Aβ increases ROS levels, which in turn potentiate Aβ deposition (**Figure 2**) (Park et al., 2011). There is also evidence showing that Aβ can induce Nox activation and ROS production, which are responsible for the activation of multiple mechanisms involved in vascular dysfunction and decreased levels of tight junction proteins mRNA (Carrano et al., 2011). However, Nox driven ROS production does not seem to affect Aβ production in Tg2576 mice, a mouse model of AD, lacking the key Nox 2 subunit (Park et al., 2008, 2011).

Endothelial ROS overproduction upregulates the endothelial production and release of endothelin-1, a well know vasoconstrictor that appears to be increased in AD (Palmer et al., 2013). Likewise, ROS overproduction seems to be responsible for reduced ^•^NO bioavailability mainly by its fast reaction with O_2_^•-^ generating ONOO^-^ (Park et al., 2005; Tong et al., 2005) and for a decreased activity of potassium channels (Erdos et al., 2004), two major factors required for endothelial-mediated dilations in brain vasculature (Hamel et al., 2016). Moreover, Aβ-induced nitrosative stress in endothelial cells is also responsible for DNA damage, resulting in poly (ADP-ribose) polymerase (PARP) activation, ultimately leading to a large increase in intracellular Ca^2+^ through transient receptor potential melastatin-2 channels activation (**Figure 2**) (Park et al., 2014).

Reactive oxygen species overproduction also interferes with HIF-1α reducing its expression and activity which will restrain the stimulus to promote angiogenesis and new vessels formation (Ogunshola and Antoniou, 2009) leading to a vicious cycle of impaired capillary perfusion, hypoxia and oxidative stress (Mamelak, 2017).

## Final Remarks

Recognizing vascular ROS production as one of the main causes of BBB dysfunction in aging and dementia, particularly in AD, raises the prospect that pharmacotherapy targeting the major ROS producers, namely mitochondria and/or Nox, might open a new venue to stop or, at least, slow dementia progression (Sweeney et al., 2015). Indeed, relying on the overwhelming evidence that mitochondrial dysfunction could be in the genesis of neurovascular dysfunction, several molecules with potential therapeutic effects were designed to target ROS, boost mitochondrial function and decrease free radical production and oxidative damage (Tong et al., 2005; Hamel et al., 2008; Moon et al., 2014) in an attempt to improve neurovascular health. Some of those molecules ameliorated the cognitive performance of mouse models of cerebrovascular disease (Tong et al., 2005). It was observed that the steroid hormone 17β-estradiol (estrogen) modulates the expression of several transcriptional regulators causing a decrease in PGC-1α, and an increase in PGC-1β, PGC-1-related coactivator (PRC), nuclear respiratory factor 1 (NRF-1) and mitochondrial transcription factor A (TFAM), which protect cerebral blood vessels (**Table 1**) (Kemper et al., 2014). These protective effects were due to increased mitochondrial biogenesis and antioxidant enzymes (Kemper et al., 2014). However, the use of antioxidant therapy in cerebrovascular disease still needs validation. Indeed, several promising approaches failed in clinical trials (Tarantini et al., 2017b), opening an intense debate about the reasons for the failure of those molecules in humans. Merely as an example, the use of tempol showed promising results in improving vasodilator responses of cerebral arterioles in aged APP transgenic mice with CAA (Han et al., 2015) but so far there is no evidence of its efficacy in clinical trials. Furthermore, two major concerns arise from the use of antioxidants as therapeutic approaches: (1) physiological ROS concentrations are important in normal cell functioning and the use of antioxidants may interfere with ROS signaling and (2) the concentrations of antioxidants revealed to be suboptimal doses in certain situations. Indeed, it is known now that supra-physiological concentrations are required to compete with the constant reaction that usually occurs between O_2_^•-^ and ^•^NO (Drummond et al., 2011). Large part of the studies failed to prove that antioxidants reach the vasculature at appropriate/therapeutic concentrations (De Silva and Miller, 2016).

**Table 1:** Antioxidant based therapies.

Molecular target	Compound	Mechanism of action	Reference
Mitochondria	17β-estradiol	↑Mitochondrial biogenesis	Kemper et al., 2014
		↑Antioxidant enzymes	
	Mitotempo	↑Mitochondrial function	Pung et al., 2012;
		↓Vascular O_2_^-^	Kizhakekuttu et al., 2012;
		↑Vascular NO production	Carvalho et al., 2014;
			Tang et al., 2014
	MitoQ	↓Oxidative stress	Ghosh et al., 2010;
		↓Endothelial dysfunction	Rodriguez-Cuenca et al., 2010;
		↓Leukocytes adhesion	Smith and Murphy, 2011;
			Gioscia-Ryan et al., 2014
NADPH oxidases	Genetic deletion of Nox 2	Improve cerebral vessels functioning	Park et al., 2007, 2008
	Apocynin	↓Nox-derived ROS production	Mayhan et al., 2008
	Diphenyleneiodonium	↑eNOS-driven reactivity	
	chloride		
	Fulvene–5	Inhibit extracellular Nox domains	Perry et al., 2006;
	triphenylmethane derivatives	(under investigation)	Bhandarkar et al., 2009;
	grindelic acid		Gianni et al., 2010;
	ML171		Munson et al., 2012;
			Kofler et al., 2013

To overcome the failure of the traditional antioxidants, researchers are developing new specific-targeted antioxidants. However, evidence about the specific-target antioxidants effects in endothelial cells is scarce. Moreover, mitochondria-targeted antioxidants decrease the dose required and limit toxic side effects rendering them a promising therapeutic approach (Smith and Murphy, 2011). It was observed that MitoTempo, a mitochondria-targeted antioxidant, was able to counteract Aβ-induced damage under hyperglycemic conditions in rat and mouse primary cultures of endothelial cells (Carvalho et al., 2014). Additionally, it was shown that MitoTempo was able to improve mitochondrial function and coronary collateral growth after ischemia/reperfusion in Zucker fatty rats (**Table 1**) (Pung et al., 2012). Recent studies also showed that MitoTempo was able to decrease vascular O_2_^•-^ and increase vascular ^•^NO production improving endothelial-dependent relaxation in angiotensin II-induced hypertensive C57Bl/6 mice (Tang et al., 2014). It was also observed that MitoTempo improves endothelial function and reduces mitochondrial O_2_^•-^ levels in subcutaneous arterioles isolated from type 2 diabetic patients (**Table 1**) (Kizhakekuttu et al., 2012). Moreover, MitoQ, another mitochondria-targeted antioxidant was able to reduce oxidative stress, without causing adverse effects, on wild-type mice (Rodriguez-Cuenca et al., 2010; Smith et al., 2011) and animal models of neurodegenerative diseases such as AD and PD (Ghosh et al., 2010; Manczak et al., 2010). MitoQ was able to improve age-related arterial endothelial dysfunction in C57BL/6 mice (**Table 1**) (Gioscia-Ryan et al., 2014). Furthermore, MitoQ has been shown to prevent cocaine-induced cardiac dysfunction in Wistar rats (Vergeade et al., 2010). However, the use of mitochondrial-directed drugs should take in account two major limitations: first, the lack of organ-specificity, leading to a greater accumulation in mitochondria-rich tissues; and second the typically used chemicals tend to accumulate in the matrix and the matrix-facing surface of the inner mitochondrial membrane, over other important mitochondrial compartments (Leitao-Rocha et al., 2015). The majority of mitochondria-targeted compounds were already able to identify and get ahead of these limiting factors in human clinical trials, defining the amount of the compound that can be administered safely. For example, MitoQ, was already developed as a pharmaceutical by Antipodean Pharmaceuticals Inc. (Smith et al., 2008) and clinical trials are running, namely in type 2 diabetic patients, where MitoQ treatment was able to decrease ROS levels and significantly reduced the adhesion of leukocytes to endothelial cells in type 2 diabetic individuals (Escribano-Lopez et al., 2016).

Likewise, approaches designed to target Nox-derived ROS overproduction are also under intense scrutiny. Indeed, the genetic deletion or inhibition of Nox2 seems effective in improving cerebral vessels functioning in aged APP mice (Park et al., 2007, 2008). Apocynin and diphenyleniodonium are widely used to ameliorate Nox-related cerebrovascular dysfunction, however, both compounds present non-specific effects and cannot be used in the clinic (**Table 1**) (De Silva and Miller, 2016). In this line, several attempts are being made to synthesize new Nox inhibitors including fulvene-5, triphenylmethane derivatives, grindelic acid, and ML171 (**Table 1**) (De Silva and Miller, 2016). However, results are scarce and further studies are needed to demonstrate that those compounds are potential therapeutics for the treatment of neurodegenerative conditions characterized by oxidative stress-associated vascular alterations.

In sum, despite the existing information about the role of cerebrovascular oxidative stress in neurodegenerative conditions, a long way is still ahead to clarify the mechanisms of age-associated vascular damage and subsequent neurodegenerative conditions. New information is crucial for the design of more effective therapeutic strategies.

## Author Contributions

CC performed literature search and wrote the paper. PM provided a critical revision of the paper.

## Conflict of Interest Statement

The authors declare that the research was conducted in the absence of any commercial or financial relationships that could be construed as a potential conflict of interest. The handling Editor declared a shared affiliation, though no other collaboration, with the authors.

## References

[B1] Agarwal R. Shukla G. S. (1999). Potential role of cerebral glutathione in the maintenance of blood-brain barrier integrity in rat. *Neurochem. Res.* 24 1507–1514. 10.1023/A:1021191729865 10591399

[B2] Aghajanian A. Wittchen E. S. Campbell S. L. Burridge K. (2009). Direct activation of RhoA by reactive oxygen species requires a redox-sensitive motif. *PLoS One* 4:e8045. 10.1371/journal.pone.0008045 19956681 PMC2778012

[B3] Al-Bachari S. Vidyasagar R. Emsley H. C. Parkes L. M. (2017). Structural and physiological neurovascular changes in idiopathic Parkinson’s disease and its clinical phenotypes. *J. Cereb. Blood Flow Metab.* 37 3409–3421. 10.1177/0271678X16688919 28112022 PMC5624390

[B4] Alfadda A. A. Sallam R. M. (2012). Reactive oxygen species in health and disease. *J. Biomed. Biotechnol.* 2012:936486. 10.1155/2012/936486 22927725 PMC3424049

[B5] Allen C. L. Bayraktutan U. (2009). Oxidative stress and its role in the pathogenesis of ischaemic stroke. *Int. J. Stroke* 4 461–470. 10.1111/j.1747-4949.2009.00387.x 19930058

[B6] Alyautdin R. Khalin I. Nafeeza M. I. Haron M. H. Kuznetsov D. (2014). Nanoscale drug delivery systems and the blood-brain barrier. *Int. J. Nanomedicine* 9 795–811. 10.2147/IJN.S52236 24550672 PMC3926460

[B7] Armulik A. Genove G. Mae M. Nisancioglu M. H. Wallgard E. Niaudet C. (2010). Pericytes regulate the blood-brain barrier. *Nature* 468 557–561. 10.1038/nature09522 20944627

[B8] Austin S. A. Santhanam A. V. d’Uscio L. V. Katusic Z. S. (2015). Regional heterogeneity of cerebral microvessels and brain susceptibility to oxidative stress. *PLoS One* 10:e0144062. 10.1371/journal.pone.0144062 26629821 PMC4668095

[B9] Baloyannis S. J. Baloyannis I. S. (2012). The vascular factor in Alzheimer’s disease: a study in Golgi technique and electron microscopy. *J. Neurol. Sci.* 322 117–121. 10.1016/j.jns.2012.07.010 22857991

[B10] Bell R. D. Zlokovic B. V. (2009). Neurovascular mechanisms and blood-brain barrier disorder in Alzheimer’s disease. *Acta Neuropathol.* 118 103–113. 10.1007/s00401-009-0522-3 19319544 PMC2853006

[B11] Bhandarkar S. S. Jaconi M. Fried L. E. Bonner M. Y. Lefkove B. Govindarajan B. (2009). Fulvene-5 potently inhibits NADPH oxidase 4 and blocks the growth of endothelial tumors in mice. *J. Clin. Invest.* 119 2359–2365. 10.1172/JCI33877 19620773 PMC2719922

[B12] Brandes R. P. Viedt C. Nguyen K. Beer S. Kreuzer J. Busse R. (2001). Thrombin-induced MCP-1 expression involves activation of the p22phox-containing NADPH oxidase in human vascular smooth muscle cells. *Thromb. Haemost.* 85 1104–1110. 10.1055/s-0037-1615970 11434692

[B13] Brown G. C. Borutaite V. (2001). Nitric oxide, mitochondria, and cell death. *IUBMB Life* 52 189–195. 10.1080/15216540152845993 11798032

[B14] Busija D. W. Katakam P. V. (2014). Mitochondrial mechanisms in cerebral vascular control: shared signaling pathways with preconditioning. *J. Vasc. Res.* 51 175–189. 10.1159/000360765 24862206 PMC4149841

[B15] Busija D. W. Rutkai I. Dutta S. Katakam P. V. (2016). Role of mitochondria in cerebral vascular function: energy production, cellular protection, and regulation of vascular tone. *Compr. Physiol.* 6 1529–1548. 10.1002/cphy.c150051 27347901 PMC12983452

[B16] Cadenas E. Davies K. J. (2000). Mitochondrial free radical generation, oxidative stress, and aging. *Free Radic. Biol. Med.* 29 222–230. 10.1016/S0891-5849(00)00317-8 11035250

[B17] Camici G. G. Savarese G. Akhmedov A. Luscher T. F. (2015). Molecular mechanism of endothelial and vascular aging: implications for cardiovascular disease. *Eur. Heart J.* 36 3392–3403. 10.1093/eurheartj/ehv587 26543043

[B18] Campos-Pena V. Toral-Rios D. Becerril-Perez F. Sanchez-Torres C. Delgado-Namorado Y. Torres-Ossorio E. (2017). Metabolic syndrome as a risk factor for Alzheimer’s disease: is abeta a crucial factor in both pathologies? *Antioxid. Redox Signal.* 26 542–560. 10.1089/ars.2016.6768 27368351

[B19] Candeias E. Duarte A. I. Sebastiao I. Fernandes M. A. Placido A. I. Carvalho C. (2017). Middle-aged diabetic females and males present distinct susceptibility to Alzheimer disease-like pathology. *Mol. Neurobiol.* 54 6471–6489. 10.1007/s12035-016-0155-1 27730513

[B20] Caplan L. R. Biller J. Leary M. C. Lo E. H. Thomas A. J. Yenari M. (2017). *Primer on Cerebrovascular Diseases.* Amsterdam: Elsevier Science.

[B21] Carrano A. Hoozemans J. J. van der Vies S. M. Rozemuller A. J. van Horssen J. de Vries H. E. (2011). Amyloid Beta induces oxidative stress-mediated blood-brain barrier changes in capillary amyloid angiopathy. *Antioxid. Redox Signal.* 15 1167–1178. 10.1089/ars.2011.3895 21294650

[B22] Carvalho C. Cardoso S. Correia S. C. Santos R. X. Santos M. S. Baldeiras I. (2012). Metabolic alterations induced by sucrose intake and Alzheimer’s disease promote similar brain mitochondrial abnormalities. *Diabetes* 61 1234–1242. 10.2337/db11-1186 22427376 PMC3331754

[B23] Carvalho C. Correia S. Santos R. Cardoso S. Santos M. Moreira P. (2010). “Diabetes, mitochondria and brain endothelium dysfunction: a dangerous triad for neurodegeneration?,” in *Mitochondria: Structure, Functions and Dysfunctions*, ed. Svensson O. (Hauppauge, NY: Nova Science Publishers, Inc.), 561–577.

[B24] Carvalho C. Correia S. C. Cardoso S. Placido A. I. Candeias E. Duarte A. I. (2015). The role of mitochondrial disturbances in Alzheimer, Parkinson and Huntington diseases. *Expert Rev. Neurother.* 15 867–884. 10.1586/14737175.2015.1058160 26092668

[B25] Carvalho C. Katz P. S. Dutta S. Katakam P. V. Moreira P. I. Busija D. W. (2014). Increased susceptibility to amyloid-beta toxicity in rat brain microvascular endothelial cells under hyperglycemic conditions. *J. Alzheimers Dis.* 38 75–83. 10.3233/JAD-130464 23948922 PMC4570500

[B26] Carvalho C. Machado N. Mota P. Correia S. Cardoso S. Santos R. (2013). Type 2 diabetic and Alzheimer’s disease mice present similar behavioral, cognitive and vascular anomalies. *J. Alzheimer Dis.* 35 623–635. 10.3233/JAD-130005 23478310

[B27] Chan D. C. (2006). Mitochondria: dynamic organelles in disease, aging, and development. *Cell* 125 1241–1252. 10.1016/j.cell.2006.06.010 16814712

[B28] Chen Q. Wang Q. Zhu J. Xiao Q. Zhang L. (2017). Reactive oxygen species: key regulators in vascular health and diseases. *Br. J. Pharmacol.* 175 1279–1292. 10.1111/bph.13828 28430357 PMC5867026

[B29] Christov-Moore L. Simpson E. A. Coude G. Grigaityte K. Iacoboni M. Ferrari P. F. (2014). Empathy: gender effects in brain and behavior. *Neurosci. Biobehav. Rev.* 46(Pt 4), 604–627. 10.1016/j.neubiorev.2014.09.001 25236781 PMC5110041

[B30] Correia S. C. Carvalho C. Cardoso S. Santos R. X. Santos M. S. Oliveira C. R. (2010). Mitochondrial preconditioning: a potential neuroprotective strategy. *Front. Aging Neurosci.* 2:138 10.3389/fnagi.2010.00138 PMC2936931 20838473

[B31] Correia S. C. Santos R. X. Cardoso S. M. Santos M. S. Oliveira C. R. Moreira P. I. (2012). Cyanide preconditioning protects brain endothelial and NT2 neuron-like cells against glucotoxicity: role of mitochondrial reactive oxygen species and HIF-1alpha. *Neurobiol. Dis.* 45 206–218. 10.1016/j.nbd.2011.08.005 21854848

[B32] Csiszar A. Labinskyy N. Orosz Z. Xiangmin Z. Buffenstein R. Ungvari Z. (2007). Vascular aging in the longest-living rodent, the naked mole rat. *Am. J. Physiol. Heart Circ. Physiol.* 293 H919–H927. 10.1152/ajpheart.01287.2006 17468332

[B33] Culic O. Gruwel M. L. Schrader J. (1997). Energy turnover of vascular endothelial cells. *Am. J. Physiol.* 273(1 Pt 1), C205–C213. 10.1152/ajpcell.1997.273.1.C205 9252458

[B34] Daneman R. Prat A. (2015). The blood-brain barrier. *Cold Spring Harb. Perspect. Biol.* 7:a020412. 10.1101/cshperspect.a020412 25561720 PMC4292164

[B35] de Almeida A. Ribeiro T. P. de Medeiros I. A. (2017). Aging: molecular pathways and implications on the cardiovascular system. *Oxid. Med. Cell. Longev.* 2017:7941563. 10.1155/2017/7941563 28874954 PMC5569936

[B36] De Keulenaer G. W. Alexander R. W. Ushio-Fukai M. Ishizaka N. Griendling K. K. (1998). Tumour necrosis factor alpha activates a p22phox-based NADH oxidase in vascular smooth muscle. *Biochem. J.* 329(Pt 3), 653–657. 10.1042/bj3290653 9445395 PMC1219089

[B37] De Silva T. M. Faraci F. M. (2016). Microvascular dysfunction and cognitive impairment. *Cell. Mol. Neurobiol.* 36 241–258. 10.1007/s10571-015-0308-1 26988697 PMC4846472

[B38] De Silva T. M. Miller A. A. (2016). Cerebral small vessel disease: targeting oxidative stress as a novel therapeutic strategy? *Front. Pharmacol.* 7:61. 10.3389/fphar.2016.00061 27014073 PMC4794483

[B39] De Strooper B. Karran E. (2016). The cellular phase of Alzheimer’s disease. *Cell* 164 603–615. 10.1016/j.cell.2015.12.056 26871627

[B40] Didion S. P. Faraci F. M. (2002). Effects of NADH and NADPH on superoxide levels and cerebral vascular tone. *Am. J. Physiol. Heart Circ. Physiol.* 282 H688–H695. 10.1152/ajpheart.00576.2001 11788419

[B41] Dikalov S. I. Nazarewicz R. R. Bikineyeva A. Hilenski L. Lassegue B. Griendling K. K. (2014). Nox2-induced production of mitochondrial superoxide in angiotensin II-mediated endothelial oxidative stress and hypertension. *Antioxid. Redox Signal.* 20 281–294. 10.1089/ars.2012.4918 24053613 PMC3887459

[B42] Dikalov S. I. Dikalova A. E. Bikineyeva A. T. Schmidt H. H. Harrison D. G. Griendling K. K. (2008). Distinct roles of Nox1 and Nox4 in basal and angiotensin II-stimulated superoxide and hydrogen peroxide production. *Free Radic. Biol. Med.* 45 1340–1351. 10.1016/j.freeradbiomed.2008.08.013 18760347 PMC2630771

[B43] Ding X. Zhang M. Gu R. Xu G. Wu H. (2017). Activated microglia induce the production of reactive oxygen species and promote apoptosis of co-cultured retinal microvascular pericytes. *Graefes Arch. Clin. Exp. Ophthalmol.* 255 777–788. 10.1007/s00417-016-3578-5 28074262

[B44] Dromparis P. Michelakis E. D. (2012). Mitochondria in vascular health and disease. *Annu. Rev. Physiol.* 75 95–126. 10.1146/annurev-physiol-030212-183804 23157555

[B45] Drummond G. R. Selemidis S. Griendling K. K. Sobey C. G. (2011). Combating oxidative stress in vascular disease: NADPH oxidases as therapeutic targets. *Nat. Rev. Drug Discov.* 10 453–471. 10.1038/nrd3403 21629295 PMC3361719

[B46] Drummond G. R. Sobey C. G. (2014). Endothelial NADPH oxidases: which NOX to target in vascular disease? *Trends Endocrinol. Metab.* 25 452–463. 10.1016/j.tem.2014.06.012 25066192

[B47] Enciu A. M. Gherghiceanu M. Popescu B. O. (2013). Triggers and effectors of oxidative stress at blood-brain barrier level: relevance for brain ageing and neurodegeneration. *Oxid. Med. Cell. Longev.* 2013:297512. 10.1155/2013/297512 23533687 PMC3606793

[B48] Erdos B. Snipes J. A. Miller A. W. Busija D. W. (2004). Cerebrovascular dysfunction in Zucker obese rats is mediated by oxidative stress and protein kinase C. *Diabetes* 53 1352–1359. 10.2337/diabetes.53.5.1352 15111506

[B49] Escribano-Lopez I. Diaz-Morales N. Rovira-Llopis S. de Maranon A. M. Orden S. Alvarez A. (2016). The mitochondria-targeted antioxidant MitoQ modulates oxidative stress, inflammation and leukocyte-endothelium interactions in leukocytes isolated from type 2 diabetic patients. *Redox Biol.* 10 200–205. 10.1016/j.redox.2016.10.017 27810734 PMC5094376

[B50] Faraci F. M. (2017). Disease highlights the cellular diversity of neurovascular units: sign in stranger. *Circ. Res.* 121 203–205. 10.1161/CIRCRESAHA.117.311386 28729449 PMC5539904

[B51] Faraci F. M. Sobey C. G. (1998). Role of potassium channels in regulation of cerebral vascular tone. *J. Cereb. Blood Flow Metab.* 18 1047–1063. 10.1097/00004647-199810000-00001 9778181

[B52] Faraco G. Moraga A. Moore J. Anrather J. Pickel V. M. Iadecola C. (2013). Circulating endothelin-1 alters critical mechanisms regulating cerebral microcirculation. *Hypertension* 62 759–766. 10.1161/HYPERTENSIONAHA.113.01761 23959559 PMC3874144

[B53] Faraco G. Sugiyama Y. Lane D. Garcia-Bonilla L. Chang H. Santisteban M. M. (2016). Perivascular macrophages mediate the neurovascular and cognitive dysfunction associated with hypertension. *J. Clin. Invest.* 126 4674–4689. 10.1172/JCI86950 27841763 PMC5127678

[B54] Freeman L. R. Keller J. N. (2012). Oxidative stress and cerebral endothelial cells: regulation of the blood-brain-barrier and antioxidant based interventions. *Biochim. Biophys. Acta* 1822 822–829. 10.1016/j.bbadis.2011.12.009 22206999 PMC3412391

[B55] Gallart-Palau X. Lee B. S. Adav S. S. Qian J. Serra A. Park J. E. (2016). Gender differences in white matter pathology and mitochondrial dysfunction in Alzheimer’s disease with cerebrovascular disease. *Mol. Brain* 9:27. 10.1186/s13041-016-0205-7 26983404 PMC4794845

[B56] Ghosh A. Chandran K. Kalivendi S. V. Joseph J. Antholine W. E. Hillard C. J. (2010). Neuroprotection by a mitochondria-targeted drug in a Parkinson’s disease model. *Free Radic. Biol. Med.* 49 1674–1684. 10.1016/j.freeradbiomed.2010.08.028 20828611 PMC4020411

[B57] Gianni D. Taulet N. Zhang H. DerMardirossian C. Kister J. Martinez L. (2010). A novel and specific NADPH oxidase-1 (Nox1) small-molecule inhibitor blocks the formation of functional invadopodia in human colon cancer cells. *ACS Chem. Biol.* 5 981–993. 10.1021/cb100219n 20715845 PMC2955773

[B58] Giordano G. Tait L. Furlong C. E. Cole T. B. Kavanagh T. J. Costa L. G. (2013). Gender differences in brain susceptibility to oxidative stress are mediated by levels of paraoxonase-2 expression. *Free Radic. Biol. Med.* 58 98–108. 10.1016/j.freeradbiomed.2013.01.019 23376469 PMC3622778

[B59] Gioscia-Ryan R. A. LaRocca T. J. Sindler A. L. Zigler M. C. Murphy M. P. Seals D. R. (2014). Mitochondria-targeted antioxidant (MitoQ) ameliorates age-related arterial endothelial dysfunction in mice. *J. Physiol.* 592 2549–2561. 10.1113/jphysiol.2013.268680 24665093 PMC4080937

[B60] Girouard H. Iadecola C. (2006). Neurovascular coupling in the normal brain and in hypertension, stroke, and Alzheimer disease. *J. Appl. Physiol.* 100 328–335. 10.1152/japplphysiol.00966.2005 16357086

[B61] Girouard H. Park L. Anrather J. Zhou P. Iadecola C. (2006). Angiotensin II attenuates endothelium-dependent responses in the cerebral microcirculation through nox-2-derived radicals. *Arterioscler. Thromb. Vasc. Biol.* 26 826–832. 10.1161/01.ATV.0000205849.22807.6e 16439707

[B62] Girouard H. Park L. Anrather J. Zhou P. Iadecola C. (2007). Cerebrovascular nitrosative stress mediates neurovascular and endothelial dysfunction induced by angiotensin II. *Arterioscler. Thromb. Vasc. Biol.* 27 303–309. 10.1161/01.ATV.0000253885.41509.25 17138940

[B63] Goetz M. E. Luch A. (2008). Reactive species: a cell damaging rout assisting to chemical carcinogens. *Cancer Lett.* 266 73–83. 10.1016/j.canlet.2008.02.035 18367325

[B64] Gordon G. R. Mulligan S. J. MacVicar B. A. (2007). Astrocyte control of the cerebrovasculature. *Glia* 55 1214–1221. 10.1002/glia.20543 17659528

[B65] Gorlach A. Diebold I. Schini-Kerth V. B. Berchner-Pfannschmidt U. Roth U. Brandes R. P. (2001). Thrombin activates the hypoxia-inducible factor-1 signaling pathway in vascular smooth muscle cells: role of the p22(phox)-containing NADPH oxidase. *Circ. Res.* 89 47–54. 10.1161/hh1301.092678 11440977

[B66] Grochowski C. Litak J. Kamieniak P. Maciejewski R. (2018). Oxidative stress in cerebral small vessel disease. Role of reactive species. *Free Radic. Res.* 52 1–13. 10.1080/10715762.2017.1402304 29166803

[B67] Halliwell B. (2001). Role of free radicals in the neurodegenerative diseases: therapeutic implications for antioxidant treatment. *Drugs Aging* 18 685–716. 10.2165/00002512-200118090-00004 11599635

[B68] Hamel E. Nicolakakis N. Aboulkassim T. Ongali B. Tong X. K. (2008). Oxidative stress and cerebrovascular dysfunction in mouse models of Alzheimer’s disease. *Exp. Physiol.* 93 116–120. 10.1113/expphysiol.2007.038729 17911359

[B69] Hamel E. Royea J. Ongali B. Tong X. K. (2016). Neurovascular and cognitive failure in Alzheimer’s disease: benefits of cardiovascular therapy. *Cell. Mol. Neurobiol.* 36 219–232. 10.1007/s10571-015-0285-4 26993506 PMC11482419

[B70] Han B. H. Zhou M. L. Johnson A. W. Singh I. Liao F. Vellimana A. K. (2015). Contribution of reactive oxygen species to cerebral amyloid angiopathy, vasomotor dysfunction, and microhemorrhage in aged Tg2576 mice. *Proc. Natl. Acad. Sci. U.S.A.* 112 E881–E890. 10.1073/pnas.1414930112 25675483 PMC4345564

[B71] Harman D. (1956). Aging: a theory based on free radical and radiation chemistry. *J. Gerontol.* 11 298–300. 10.1093/geronj/11.3.298 13332224

[B72] Helmcke I. Heumuller S. Tikkanen R. Schroder K. Brandes R. P. (2009). Identification of structural elements in Nox1 and Nox4 controlling localization and activity. *Antioxid. Redox Signal.* 11 1279–1287. 10.1089/ARS.2008.2383 19061439

[B73] Hopkins N. D. Stratton G. Tinken T. M. Ridgers N. D. Graves L. E. McWhannell N. (2011). Seasonal reduction in physical activity and flow-mediated dilation in children. *Med. Sci. Sports Exerc.* 43 232–238. 10.1249/MSS.0b013e3181ebe90e 20581722

[B74] Hu W. T. Wang Z. Lee V. M. Trojanowski J. Q. Detre J. A. Grossman M. (2010). Distinct cerebral perfusion patterns in FTLD and AD. *Neurology* 75 881–888. 10.1212/WNL.0b013e3181f11e35 20819999 PMC2938974

[B75] Hunter J. M. Kwan J. Malek-Ahmadi M. Maarouf C. L. Kokjohn T. A. Belden C. (2012). Morphological and pathological evolution of the brain microcirculation in aging and Alzheimer’s disease. *PLoS One* 7:e36893. 10.1371/journal.pone.0036893 22615835 PMC3353981

[B76] Iadecola C. (2013). The pathobiology of vascular dementia. *Neuron* 80 844–866. 10.1016/j.neuron.2013.10.008 24267647 PMC3842016

[B77] Ingalhalikar M. Smith A. Parker D. Satterthwaite T. D. Elliott M. A. Ruparel K. (2014). Sex differences in the structural connectome of the human brain. *Proc. Natl. Acad. Sci. U.S.A.* 111 823–828. 10.1073/pnas.1316909110 24297904 PMC3896179

[B78] Jackman K. A. Miller A. A. Drummond G. R. Sobey C. G. (2009). Importance of NOX1 for angiotensin II-induced cerebrovascular superoxide production and cortical infarct volume following ischemic stroke. *Brain Res.* 1286 215–220. 10.1016/j.brainres.2009.06.056 19559686

[B79] Jansen F. Yang X. Franklin B. S. Hoelscher M. Schmitz T. Bedorf J. (2013). High glucose condition increases NADPH oxidase activity in endothelial microparticles that promote vascular inflammation. *Cardiovasc. Res.* 98 94–106. 10.1093/cvr/cvt013 23341580

[B80] Jellinger K. A. Mitter-Ferstl E. (2003). The impact of cerebrovascular lesions in Alzheimer disease–a comparative autopsy study. *J. Neurol.* 250 1050–1055. 10.1007/s00415-003-0142-0 14504965

[B81] Kahles T. Luedike P. Endres M. Galla H. J. Steinmetz H. Busse R. (2007). NADPH oxidase plays a central role in blood-brain barrier damage in experimental stroke. *Stroke* 38 3000–3006. 10.1161/STROKEAHA.107.489765 17916764

[B82] Kemper M. F. Stirone C. Krause D. N. Duckles S. P. Procaccio V. (2014). Genomic and non-genomic regulation of PGC1 isoforms by estrogen to increase cerebral vascular mitochondrial biogenesis and reactive oxygen species protection. *Eur. J. Pharmacol.* 723 322–329. 10.1016/j.ejphar.2013.11.009 24275351 PMC4028038

[B83] Kivipelto M. Laakso M. P. Tuomilehto J. Nissinen A. Soininen H. (2002). Hypertension and hypercholesterolaemia as risk factors for Alzheimer’s disease: potential for pharmacological intervention. *CNS Drugs* 16 435–444. 10.2165/00023210-200216070-00001 12056919

[B84] Kizhakekuttu T. J. Wang J. Dharmashankar K. Ying R. Gutterman D. D. Vita J. A. (2012). Adverse alterations in mitochondrial function contribute to type 2 diabetes mellitus-related endothelial dysfunction in humans. *Arterioscler. Thromb. Vasc. Biol.* 32 2531–2539. 10.1161/ATVBAHA.112.256024 22879582 PMC3570053

[B85] Kling M. A. Trojanowski J. Q. Wolk D. A. Lee V. M. Arnold S. E. (2013). Vascular disease and dementias: paradigm shifts to drive research in new directions. *Alzheimers Dement.* 9 76–92. 10.1016/j.jalz.2012.02.007 23183137 PMC3640817

[B86] Kluge M. A. Fetterman J. L. Vita J. A. (2013). Mitochondria and endothelial function. *Circ. Res.* 112 1171–1188. 10.1161/CIRCRESAHA.111.300233 23580773 PMC3700369

[B87] Konior A. Schramm A. Czesnikiewicz-Guzik M. Guzik T. J. (2014). NADPH oxidases in vascular pathology. *Antioxid. Redox Signal.* 20 2794–2814. 10.1089/ars.2013.5607 24180474 PMC4026218

[B88] Kubíková T. Kochova P. Tomasek P. Witter K. Tonar Z. (2017). Numerical and length densities of microvessels in the human brain: correlation with preferential orientation of microvessels in the cerebral cortex, subcortical grey matter and white matter, pons and cerebellum. *J. Chem. Neuroanat.* 88 22–32. 10.1016/j.jchemneu.2017.11.005 29113946

[B89] Landmesser U. Dikalov S. Price S. R. McCann L. Fukai T. Holland S. M. (2003). Oxidation of tetrahydrobiopterin leads to uncoupling of endothelial cell nitric oxide synthase in hypertension. *J. Clin. Invest.* 111 1201–1209. 10.1172/JCI14172 12697739 PMC152929

[B90] Lee H. Pienaar I. S. (2014). Disruption of the blood-brain barrier in Parkinson’s disease: curse or route to a cure? *Front. Biosci.* 19 272–280. 10.2741/4206 24389183

[B91] Lee J. M. Shih A. Y. Murphy T. H. Johnson J. A. (2003). NF-E2-related factor-2 mediates neuroprotection against mitochondrial complex I inhibitors and increased concentrations of intracellular calcium in primary cortical neurons. *J. Biol. Chem.* 278 37948–37956. 10.1074/jbc.M305204200 12842875

[B92] Leitao-Rocha A. Guedes-Dias P. Pinho B. R. Oliveira J. M. (2015). Trends in mitochondrial therapeutics for neurological disease. *Curr. Med. Chem.* 22 2458–2467. 10.2174/0929867322666150209160317 25666789

[B93] Li J. Li W. Su J. Liu W. Altura B. T. Altura B. M. (2003). Hydrogen peroxide induces apoptosis in cerebral vascular smooth muscle cells: possible relation to neurodegenerative diseases and strokes. *Brain Res. Bull.* 62 101–106. 10.1016/j.brainresbull.2003.08.011 14638383

[B94] Lin C. Y. Hsu Y. H. Lin M. H. Yang T. H. Chen H. M. Chen Y. C. (2013). Neurovascular abnormalities in humans and mice with Huntington’s disease. *Exp. Neurol.* 250 20–30. 10.1016/j.expneurol.2013.08.019 24036415

[B95] Lin S. L. Liao A. Y. Yeh S. J. Lin J. Y. (2015). The analysis of cardio-respiratory signals and cerebral autoregulation based on CO_2_ reactivity with healthy subjects and Parkinson’s patients. *Technol. Health Care* 24(Suppl. 1), S195–S203. 10.3233/THC-151069 26684566

[B96] Liu Y. Zhao H. Li H. Kalyanaraman B. Nicolosi A. C. Gutterman D. D. (2003). Mitochondrial sources of H_2_O_2_ generation play a key role in flow-mediated dilation in human coronary resistance arteries. *Circ. Res.* 93 573–580. 10.1161/01.RES.0000091261.19387.AE 12919951

[B97] Lochhead J. J. McCaffrey G. Quigley C. E. Finch J. DeMarco K. M. Nametz N. (2010). Oxidative stress increases blood-brain barrier permeability and induces alterations in occludin during hypoxia-reoxygenation. *J. Cereb. Blood Flow Metab.* 30 1625–1636. 10.1038/jcbfm.2010.29 20234382 PMC2949263

[B98] Lourenço C. F. Ledo A. Barbosa R. M. Laranjinha J. (2017). Neurovascular uncoupling in the triple transgenic model of Alzheimer’s disease: impaired cerebral blood flow response to neuronal-derived nitric oxide signaling. *Exp. Neurol.* 291 36–43. 10.1016/j.expneurol.2017.01.013 28161255

[B99] Love S. Chalmers K. Ince P. Esiri M. Attems J. Jellinger K. (2014). Development, appraisal, validation and implementation of a consensus protocol for the assessment of cerebral amyloid angiopathy in post-mortem brain tissue. *Am. J. Neurodegener. Dis.* 3 19–32. 24754000 PMC3986608

[B100] Lu L. Guo L. Gauba E. Tian J. Wang L. Tandon N. (2015). Transient cerebral ischemia promotes brain mitochondrial dysfunction and exacerbates cognitive impairments in young 5xFAD mice. *PLoS One* 10:e0144068. 10.1371/journal.pone.0144068 26632816 PMC4669173

[B101] Mamelak M. (2017). Energy and the Alzheimer brain. *Neurosci. Biobehav. Rev.* 75 297–313. 10.1016/j.neubiorev.2017.02.001 28193453

[B102] Manczak M. Mao P. Calkins M. J. Cornea A. Reddy A. P. Murphy M. P. (2010). Mitochondria-targeted antioxidants protect against amyloid-beta toxicity in Alzheimer’s disease neurons. *J. Alzheimers Dis.* 20(Suppl. 2), S609–S631. 10.3233/JAD-2010-100564 20463406 PMC3072711

[B103] Matsumoto T. Kobayashi T. Wachi H. Seyama Y. Kamata K. (2007). Vascular NAD(P)H oxidase mediates endothelial dysfunction in basilar arteries from Otsuka Long-Evans Tokushima Fatty (OLETF) rats. *Atherosclerosis* 192 15–24. 10.1016/j.atherosclerosis.2006.06.005 16831440

[B104] Matsuno K. Yamada H. Iwata K. Jin D. Katsuyama M. Matsuki M. (2005). Nox1 is involved in angiotensin II-mediated hypertension: a study in Nox1-deficient mice. *Circulation* 112 2677–2685. 10.1161/CIRCULATIONAHA.105.573709 16246966

[B105] Mayhan W. G. Arrick D. M. Sharpe G. M. Sun H. (2008). Age-related alterations in reactivity of cerebral arterioles: role of oxidative stress. *Microcirculation* 15 225–236. 10.1080/10739680701641421 18386218

[B106] Mergenthaler P. Lindauer U. Dienel G. A. Meisel A. (2013). Sugar for the brain: the role of glucose in physiological and pathological brain function. *Trends Neurosci.* 36 587–597. 10.1016/j.tins.2013.07.001 23968694 PMC3900881

[B107] Mertens S. Noll T. Spahr R. Krutzfeldt A. Piper H. M. (1990). Energetic response of coronary endothelial cells to hypoxia. *Am. J. Physiol.* 258(3 Pt 2), H689–H694. 10.1152/ajpheart.1990.258.3.H689 2316683

[B108] Mikhed Y. Daiber A. Steven S. (2015). Mitochondrial oxidative stress, mitochondrial DNA damage and their role in age-related vascular dysfunction. *Int. J. Mol. Sci.* 16 15918–15953. 10.3390/ijms160715918 26184181 PMC4519931

[B109] Miller A. A. Budzyn K. Sobey C. G. (2010a). Vascular dysfunction in cerebrovascular disease: mechanisms and therapeutic intervention. *Clin. Sci.* 119 1–17. 10.1042/CS20090649 20370718

[B110] Miller A. A. De Silva T. M. Judkins C. P. Diep H. Drummond G. R. Sobey C. G. (2010b). Augmented superoxide production by Nox2-containing NADPH oxidase causes cerebral artery dysfunction during hypercholesterolemia. *Stroke* 41 784–789. 10.1161/STROKEAHA.109.575365 20167907

[B111] Miller A. A. Drummond G. R. De Silva T. M. Mast A. E. Hickey H. Williams J. P. (2009). NADPH oxidase activity is higher in cerebral versus systemic arteries of four animal species: role of Nox2. *Am. J. Physiol. Heart Circ. Physiol.* 296 H220–H225. 10.1152/ajpheart.00987.2008 19028794

[B112] Miller A. A. Drummond G. R. Schmidt H. H. Sobey C. G. (2005). NADPH oxidase activity and function are profoundly greater in cerebral versus systemic arteries. *Circ. Res.* 97 1055–1062. 10.1161/01.RES.0000189301.10217.87 16210546

[B113] Montagne A. Barnes S. R. Sweeney M. D. Halliday M. R. Sagare A. P. Zhao Z. (2015). Blood-brain barrier breakdown in the aging human hippocampus. *Neuron* 85 296–302. 10.1016/j.neuron.2014.12.032 25611508 PMC4350773

[B114] Moon G. J. Kim S. J. Cho Y. H. Ryoo S. Bang O. Y. (2014). Antioxidant effects of statins in patients with atherosclerotic cerebrovascular disease. *J. Clin. Neurol.* 10 140–147. 10.3988/jcn.2014.10.2.140 24829600 PMC4017017

[B115] Mooradian A. D. (1988). Effect of aging on the blood-brain barrier. *Neurobiol. Aging* 9 31–39. 10.1016/S0197-4580(88)80013-7 3288893

[B116] Morato M. Reina-Couto M. Pinho D. Albino-Teixeira A. Sousa T. (2017). “Regulation of the renin-angiotensin-aldosterone system by reactive oxygen species,” in *Renin-Angiotensin System - Past, Present and Future*, ed. Tolekova A. (Rijeka: InTech).

[B117] Moreira P. I. Duarte A. I. Santos M. S. Rego A. C. Oliveira C. R. (2009). An integrative view of the role of oxidative stress, mitochondria and insulin in Alzheimer’s disease. *J. Alzheimers Dis.* 16 741–761. 10.3233/JAD-2009-0972 19387110

[B118] Munson J. M. Fried L. Rowson S. A. Bonner M. Y. Karumbaiah L. Diaz B. (2012). Anti-invasive adjuvant therapy with imipramine blue enhances chemotherapeutic efficacy against glioma. *Sci. Transl. Med.* 4:127ra136. 10.1126/scitranslmed.3003016 22461640

[B119] Nelson A. R. Sweeney M. D. Sagare A. P. Zlokovic B. V. (2016). Neurovascular dysfunction and neurodegeneration in dementia and Alzheimer’s disease. *Biochim. Biophys. Acta* 1862 887–900. 10.1016/j.bbadis.2015.12.016 26705676 PMC4821735

[B120] Nunomura A. Hofer T. Moreira P. I. Castellani R. J. Smith M. A. Perry G. (2009). RNA oxidation in Alzheimer disease and related neurodegenerative disorders. *Acta Neuropathol.* 118 151–166. 10.1007/s00401-009-0508-1 19271225

[B121] Ogunshola O. O. Antoniou X. (2009). Contribution of hypoxia to Alzheimer’s disease: is HIF-1alpha a mediator of neurodegeneration? *Cell. Mol. Life Sci.* 66 3555–3563. 10.1007/s00018-009-0141-0 19763399 PMC11115623

[B122] Ohta S. Meyer E. Thompson C. J. Gjedde A. (1992). Oxygen consumption of the living human brain measured after a single inhalation of positron emitting oxygen. *J. Cereb. Blood Flow Metab.* 12 179–192. 10.1038/jcbfm.1992.28 1548291

[B123] Oldendorf W. H. Cornford M. E. Brown W. J. (1976). The large apparent metabolic work capacity of the blood-brain barrier. *Trans. Am. Neurol. Assoc.* 101 157–160. 1028223

[B124] Osellame L. D. Blacker T. S. Duchen M. R. (2012). Cellular and molecular mechanisms of mitochondrial function. *Best Pract. Res. Clin. Endocrinol. Metab.* 26 711–723. 10.1016/j.beem.2012.05.003 23168274 PMC3513836

[B125] Palmer J. C. Tayler H. M. Love S. (2013). Endothelin-converting enzyme-1 activity, endothelin-1 production, and free radical-dependent vasoconstriction in Alzheimer’s disease. *J. Alzheimers Dis.* 36 577–587. 10.3233/JAD-130383 23629587

[B126] Park L. Anrather J. Girouard H. Zhou P. Iadecola C. (2007). Nox2-derived reactive oxygen species mediate neurovascular dysregulation in the aging mouse brain. *J. Cereb. Blood Flow Metab.* 27 1908–1918. 10.1038/sj.jcbfm.9600491 17429347

[B127] Park L. Anrather J. Zhou P. Frys K. Pitstick R. Younkin S. (2005). NADPH-oxidase-derived reactive oxygen species mediate the cerebrovascular dysfunction induced by the amyloid beta peptide. *J. Neurosci.* 25 1769–1777. 10.1523/JNEUROSCI.5207-04.2005 15716413 PMC6725936

[B128] Park L. Anrather J. Zhou P. Frys K. Wang G. Iadecola C. (2004). Exogenous NADPH increases cerebral blood flow through NADPH oxidase-dependent and -independent mechanisms. *Arterioscler. Thromb. Vasc. Biol.* 24 1860–1865. 10.1161/01.ATV.0000142446.75898.44 15308559

[B129] Park L. Wang G. Moore J. Girouard H. Zhou P. Anrather J. (2014). The key role of transient receptor potential melastatin-2 channels in amyloid-beta-induced neurovascular dysfunction. *Nat. Commun.* 5:5318. 10.1038/ncomms6318 25351853 PMC4283829

[B130] Park L. Wang G. Zhou P. Zhou J. Pitstick R. Previti M. L. (2011). Scavenger receptor CD36 is essential for the cerebrovascular oxidative stress and neurovascular dysfunction induced by amyloid-beta. *Proc. Natl. Acad. Sci. U.S.A.* 108 5063–5068. 10.1073/pnas.1015413108 21383152 PMC3064396

[B131] Park L. Zhou P. Pitstick R. Capone C. Anrather J. Norris E. H. (2008). Nox2-derived radicals contribute to neurovascular and behavioral dysfunction in mice overexpressing the amyloid precursor protein. *Proc. Natl. Acad. Sci. U.S.A.* 105 1347–1352. 10.1073/pnas.0711568105 18202172 PMC2234141

[B132] Pendyala S. Usatyuk P. V. Gorshkova I. A. Garcia J. G. Natarajan V. (2009). Regulation of NADPH oxidase in vascular endothelium: the role of phospholipases, protein kinases, and cytoskeletal proteins. *Antioxid. Redox Signal.* 11 841–860. 10.1089/ARS.2008.2231 18828698 PMC2850292

[B133] Perry B. N. Govindarajan B. Bhandarkar S. S. Knaus U. G. Valo M. Sturk C. (2006). Pharmacologic blockade of angiopoietin-2 is efficacious against model hemangiomas in mice. *J. Invest. Dermatol.* 126 2316–2322. 10.1038/sj.jid.5700413 16741507

[B134] Pimentel-Coelho P. M. Rivest S. (2012). The early contribution of cerebrovascular factors to the pathogenesis of Alzheimer’s disease. *Eur. J. Neurosci.* 35 1917–1937. 10.1111/j.1460-9568.2012.08126.x 22708603

[B135] Pung Y. F. Rocic P. Murphy M. P. Smith R. A. Hafemeister J. Ohanyan V. (2012). Resolution of mitochondrial oxidative stress rescues coronary collateral growth in Zucker obese fatty rats. *Arterioscler. Thromb. Vasc. Biol.* 32 325–334. 10.1161/ATVBAHA.111.241802 22155454 PMC4013346

[B136] Quintero M. Colombo S. L. Godfrey A. Moncada S. (2006). Mitochondria as signaling organelles in the vascular endothelium. *Proc. Natl. Acad. Sci. U.S.A.* 103 5379–5384. 10.1073/pnas.0601026103 16565215 PMC1459363

[B137] Ray R. Murdoch C. E. Wang M. Santos C. X. Zhang M. Alom-Ruiz S. (2011). Endothelial Nox4 NADPH oxidase enhances vasodilatation and reduces blood pressure in vivo. *Arterioscler. Thromb. Vasc. Biol.* 31 1368–1376. 10.1161/ATVBAHA.110.219238 21415386

[B138] Richter C. Kass G. E. (1991). Oxidative stress in mitochondria: its relationship to cellular Ca^2+^ homeostasis, cell death, proliferation, and differentiation. *Chem. Biol. Interact.* 77 1–23. 10.1016/0009-2797(91)90002-O 1983962

[B139] Ricquier D. Bouillaud F. (2000). Mitochondrial uncoupling proteins: from mitochondria to the regulation of energy balance. *J. Physiol.* 529(Pt 1), 3–10. 10.1111/j.1469-7793.2000.00003.x PMC2270181 11080246

[B140] Rodriguez-Cuenca S. Cocheme H.M. Logan A. Abakumova I. Prime T.A. Rose C. (2010). Consequences of long-term oral administration of the mitochondria-targeted antioxidant MitoQ to wild-type mice. *Free Radic. Biol. Med.* 48 161–172. 10.1016/j.freeradbiomed.2009.10.039 19854266

[B141] Rojo A. I. Innamorato N. G. Martin-Moreno A. M. De Ceballos M. L. Yamamoto M. Cuadrado A. (2010). Nrf2 regulates microglial dynamics and neuroinflammation in experimental Parkinson’s disease. *Glia* 58 588–598. 10.1002/glia.20947 19908287

[B142] Ronnett G. V. Ramamurthy S. Kleman A. M. Landree L. E. Aja S. (2009). AMPK in the brain: its roles in energy balance and neuroprotection. *J. Neurochem.* 109(Suppl. 1), 17–23. 10.1111/j.1471-4159.2009.05916.x 19393004 PMC2925428

[B143] Rutkai I. Dutta S. Katakam P. V. Busija D. W. (2015). Dynamics of enhanced mitochondrial respiration in female compared with male rat cerebral arteries. *Am. J. Physiol. Heart Circ. Physiol.* 309 H1490–H1500. 10.1152/ajpheart.00231.2015 26276815 PMC4666975

[B144] Sahoo S. Meijles D. N. Pagano P. J. (2016). NADPH oxidases: key modulators in aging and age-related cardiovascular diseases? *Clin. Sci.* 130 317–335. 10.1042/CS20150087 26814203 PMC4818578

[B145] Schreibelt G. Kooij G. Reijerkerk A. van Doorn R. Gringhuis S. I. van der Pol S. (2007). Reactive oxygen species alter brain endothelial tight junction dynamics via RhoA, PI3 kinase, and PKB signaling. *FASEB J.* 21 3666–3676. 10.1096/fj.07-8329com 17586731

[B146] Shah G. N. Morofuji Y. Banks W. A. Price T. O. (2013). High glucose-induced mitochondrial respiration and reactive oxygen species in mouse cerebral pericytes is reversed by pharmacological inhibition of mitochondrial carbonic anhydrases: implications for cerebral microvascular disease in diabetes. *Biochem. Biophys. Res. Commun.* 440 354–358. 10.1016/j.bbrc.2013.09.086 24076121 PMC3875343

[B147] Shah Z. A. Li R. C. Thimmulappa R. K. Kensler T. W. Yamamoto M. Biswal S. (2007). Role of reactive oxygen species in modulation of Nrf2 following ischemic reperfusion injury. *Neuroscience* 147 53–59. 10.1016/j.neuroscience.2007.02.066 17507167 PMC1961622

[B148] Sheu S. S. Nauduri D. Anders M. W. (2006). Targeting antioxidants to mitochondria: a new therapeutic direction. *Biochim. Biophys. Acta* 1762 256–265. 10.1016/j.bbadis.2005.10.007 16352423

[B149] Skoog I. Gustafson D. (2006). Update on hypertension and Alzheimer’s disease. *Neurol. Res.* 28 605–611. 10.1179/016164106X130506 16945211

[B150] Smith R. A. Adlam V. J. Blaikie F. H. Manas A. R. Porteous C. M. James A. M. (2008). Mitochondria-targeted antioxidants in the treatment of disease. *Ann. N. Y. Acad. Sci.* 1147 105–111. 10.1196/annals.1427.003 19076435

[B151] Smith R. A. Hartley R. C. Murphy M. P. (2011). Mitochondria-targeted small molecule therapeutics and probes. *Antioxid. Redox Signal.* 15 3021–3038. 10.1089/ars.2011.3969 21395490

[B152] Smith R. A. Murphy M. P. (2011). Mitochondria-targeted antioxidants as therapies. *Discov. Med.* 11 106–114. 21356165

[B153] Spahr R. Krutzfeldt A. Mertens S. Siegmund B. Piper H. M. (1989). Fatty acids are not an important fuel for coronary microvascular endothelial cells. *Mol. Cell. Biochem.* 88 59–64. 10.1007/BF00223424 2779544

[B154] Staiculescu M. C. Foote C. Meininger G. A. Martinez-Lemus L. A. (2014). The role of reactive oxygen species in microvascular remodeling. *Int. J. Mol. Sci.* 15 23792–23835. 10.3390/ijms151223792 25535075 PMC4284792

[B155] St-Amour I. Aube B. Rieux M. Cicchetti F. (2015). Targeting cerebrovascular impairments in Huntington’s disease: a novel treatment perspective. *Neurodegener. Dis. Manage.* 5 389–393. 10.2217/nmt.15.41 26517444

[B156] Steinman J. Koletar M. M. Stefanovic B. Sled J. G. (2017). 3D morphological analysis of the mouse cerebral vasculature: comparison of in vivo and ex vivo methods. *PLoS One* 12:e0186676. 10.1371/journal.pone.0186676 29053753 PMC5650181

[B157] Sun Y. Lee R. Chen Y. Collinson S. Thakor N. Bezerianos A. (2015). Progressive gender differences of structural brain networks in healthy adults: a longitudinal, diffusion tensor imaging study. *PLoS One* 10:e0118857. 10.1371/journal.pone.0118857 25742013 PMC4350987

[B158] Sweeney M. D. Sagare A. P. Zlokovic B. V. (2015). Cerebrospinal fluid biomarkers of neurovascular dysfunction in mild dementia and Alzheimer’s disease. *J. Cereb. Blood Flow Metab.* 35 1055–1068. 10.1038/jcbfm.2015.76 25899298 PMC4640280

[B159] Takac I. Schroder K. Brandes R. P. (2012). The Nox family of NADPH oxidases: friend or foe of the vascular system? *Curr. Hypertens. Rep.* 14 70–78. 10.1007/s11906-011-0238-3 22071588

[B160] Tang X. Luo Y. X. Chen H. Z. Liu D. P. (2014). Mitochondria, endothelial cell function, and vascular diseases. *Front. Physiol.* 5:175 10.3389/fphys.2014.00175 PMC4018556 24834056

[B161] Tarantini S. Fulop G. A. Kiss T. Farkas E. Zolei-Szenasi D. Galvan V. (2017a). Demonstration of impaired neurovascular coupling responses in TG2576 mouse model of Alzheimer’s disease using functional laser speckle contrast imaging. *Geroscience* 10.1007/s11357-017-9980-z [Epub ahead of print]. 28578467 PMC5636768

[B162] Tarantini S. Tran C. H. T. Gordon G. R. Ungvari Z. Csiszar A. (2017b). Impaired neurovascular coupling in aging and Alzheimer’s disease: contribution of astrocyte dysfunction and endothelial impairment to cognitive decline. *Exp. Gerontol.* 94 52–58. 10.1016/j.exger.2016.11.004 27845201 PMC5429210

[B163] Tayarani I. Chaudiere J. Lefauconnier J. M. Bourre J. M. (1987). Enzymatic protection against peroxidative damage in isolated brain capillaries. *J. Neurochem.* 48 1399–1402. 10.1111/j.1471-4159.1987.tb05677.x 3559557

[B164] Togliatto G. Lombardo G. Brizzi M. F. (2017). The future challenge of reactive oxygen species (ROS) in hypertension: from bench to bed side. *Int. J. Mol. Sci.* 18:E1988. 10.3390/ijms18091988 28914782 PMC5618637

[B165] Tong X. K. Nicolakakis N. Kocharyan A. Hamel E. (2005). Vascular remodeling versus amyloid beta-induced oxidative stress in the cerebrovascular dysfunctions associated with Alzheimer’s disease. *J. Neurosci.* 25 11165–11174. 10.1523/JNEUROSCI.4031-05.2005 16319316 PMC6725645

[B166] Toth P. Tarantini S. Tucsek Z. Ashpole N. M. Sosnowska D. Gautam T. (2014). Resveratrol treatment rescues neurovascular coupling in aged mice: role of improved cerebromicrovascular endothelial function and downregulation of NADPH oxidase. *Am. J. Physiol. Heart Circ. Physiol.* 306 H299–H308. 10.1152/ajpheart.00744.2013 24322615 PMC3920140

[B167] Ungvari Z. Labinskyy N. Gupte S. Chander P. N. Edwards J. G. Csiszar A. (2008). Dysregulation of mitochondrial biogenesis in vascular endothelial and smooth muscle cells of aged rats. *Am. J. Physiol. Heart Circ. Physiol.* 294 H2121–H2128. 10.1152/ajpheart.00012.2008 18326800

[B168] Ungvari Z. Orosz Z. Labinskyy N. Rivera A. Xiangmin Z. Smith K. (2007). Increased mitochondrial H_2_O_2_ production promotes endothelial NF-kappaB activation in aged rat arteries. *Am. J. Physiol. Heart Circ. Physiol.* 293 H37–H47. 10.1152/ajpheart.01346.2006 17416599

[B169] Vaas M. Deistung A. Reichenbach J. R. Keller A. Kipar A. Klohs J. (2017). Vascular and tissue changes of magnetic susceptibility in the mouse brain after transient cerebral ischemia. *Transl. Stroke Res.* 10.1007/s12975-017-0591-x [Epub ahead of print]. 29177950 PMC6061250

[B170] Valko M. Leibfritz D. Moncol J. Cronin M. T. Mazur M. Telser J. (2007). Free radicals and antioxidants in normal physiological functions and human disease. *Int. J. Biochem. Cell Biol.* 39 44–84. 10.1016/j.biocel.2006.07.001 16978905

[B171] Vargas M. R. Johnson J. A. (2009). The Nrf2-ARE cytoprotective pathway in astrocytes. *Expert Rev. Mol. Med.* 11:e17. 10.1017/S1462399409001094 19490732 PMC5563256

[B172] Vergeade A. Mulder P. Vendeville-Dehaudt C. Estour F. Fortin D. Ventura-Clapier R. (2010). Mitochondrial impairment contributes to cocaine-induced cardiac dysfunction: prevention by the targeted antioxidant MitoQ. *Free Radic. Biol. Med.* 49 748–756. 10.1016/j.freeradbiomed.2010.05.024 20566328

[B173] Wang M. Zhang J. Walker S. J. Dworakowski R. Lakatta E. G. Shah A. M. (2010). Involvement of NADPH oxidase in age-associated cardiac remodeling. *J. Mol. Cell. Cardiol.* 48 765–772. 10.1016/j.yjmcc.2010.01.006 20079746 PMC2877878

[B174] Wang Y. Zang Q. S. Liu Z. Wu Q. Maass D. Dulan G. (2011). Regulation of VEGF-induced endothelial cell migration by mitochondrial reactive oxygen species. *Am. J. Physiol. Cell Physiol.* 301 C695–C704. 10.1152/ajpcell.00322.2010 21653897 PMC3174570

[B175] Wenzel P. Schuhmacher S. Kienhofer J. Muller J. Hortmann M. Oelze M. (2008). Manganese superoxide dismutase and aldehyde dehydrogenase deficiency increase mitochondrial oxidative stress and aggravate age-dependent vascular dysfunction. *Cardiovasc. Res.* 80 280–289. 10.1093/cvr/cvn182 18596060 PMC3937602

[B176] Wevers N. R. de Vries H. E. (2016). Morphogens and blood-brain barrier function in health and disease. *Tissue Barriers* 4:e1090524. 10.1080/21688370.2015.1090524 27141417 PMC4836462

[B177] Yan W. Wang H. D. Hu Z. G. Wang Q. F. Yin H. X. (2008). Activation of Nrf2-ARE pathway in brain after traumatic brain injury. *Neurosci. Lett.* 431 150–154. 10.1016/j.neulet.2007.11.060 18162315

[B178] Zhang R. Ran H. H. Cai L. L. Zhu L. Sun J. F. Peng L. (2014). Simulated microgravity-induced mitochondrial dysfunction in rat cerebral arteries. *FASEB J.* 28 2715–2724. 10.1096/fj.13-245654 24604081

[B179] Zlokovic B. V. (2008). The blood-brain barrier in health and chronic neurodegenerative disorders. *Neuron* 57 178–201. 10.1016/j.neuron.2008.01.003 18215617

[B180] Zlokovic B. V. (2011). Neurovascular pathways to neurodegeneration in Alzheimer’s disease and other disorders. *Nat. Rev. Neurosci.* 12 723–738. 10.1038/nrn3114 22048062 PMC4036520

